# Retrospective clinical study on the performance and aesthetic outcome of pressed lithium disilicate restorations in posterior teeth up to 8.3 years

**DOI:** 10.1007/s00784-023-05328-0

**Published:** 2023-10-23

**Authors:** Stefanie Lindner, Iris Frasheri, Reinhard Hickel, Alexander Crispin, Andreas Kessler

**Affiliations:** 1 Department of Conservative Dentistry and Periodontology, LMU University Hospital, Ludwig-Maximilians-Universität München, Goethestr 70, 80336 Munich, Germany; 2 Department of Medical Informatics, Biometry and Epidemiology, Faculty of Medicine, Ludwig-Maximilians-Universität München, Marchioninistr 15, 81377 Munich, Germany

**Keywords:** Ceramic, Inlay, Partial crown, Survival rate, FDI criteria

## Abstract

**Objectives:**

Evaluation of cumulative survival and complication rate of monolithic lithium disilicate inlays and partial crowns performed by supervised undergraduate students up to 8.3 years of clinical service.

**Materials and methods:**

In this retrospective clinical study 143 lithium disilicate posterior restorations (IPS e.max Press) were examined according to the FDI criteria. A standardised questionnaire was used to determine patient satisfaction. The aesthetic outcome was evaluated by dentists and dental technicians using intraoral photographs. Data were descriptively analysed. Cumulative survival and success rates were calculated using Kaplan–Meier estimation.

**Results:**

The cumulative survival rate of lithium disilicate restorations was 97.5% after a mean service time of 5.9 years and 95.0% after 8.3 years. The cumulative success rate decreased from 94.4% after 5.9 years to 30.7% after 8.3 years. Repairs were required for 7 restorations (4.9%), and 5 (3.5%) were classified as failures. The results of the questionnaire indicate a high level of patient satisfaction. The subjective aesthetics were assessed more critically by dental technicians compared to dentists.

**Conclusion:**

Lithium disilicate posterior restorations survived successfully up to 8.3 years when carried out by undergraduate students.

**Clinical relevance:**

Pressed lithium disilicate glass ceramic inlays and partial crowns are reliable treatment options in posterior teeth.

**Supplementary Information:**

The online version contains supplementary material available at 10.1007/s00784-023-05328-0.

## Introduction

Lithium disilicate glass ceramic material was presented to the dental community in 2004. The lost wax technique was applied at the beginning, and in 2006, the milling version was released. Due to its good biocompatibility, high flexural strength, superior aesthetics and the possibility of bonding adhesively on enamel and dentin, the material has enjoyed increasing popularity in recent decades [1–5]. The indication of lithium disilicate ceramic covers single posterior and anterior restorations as well as 3-unit fixed bridges up to the second premolar for natural teeth and implants. The modern adhesive technique has made it possible to dispense with retentive preparation designs and stabilise the remaining tooth substance [6–8]. Due to the minimal ceramic thickness of 1 mm in load-bearing areas, the preparation design can be optimised for maximal tooth preservation, and minimally invasive treatment approaches have become established in the modern restorative dentistry [9–11].

Numerous in vitro experiments provide the required information on the properties of lithium disilicate glass ceramic such as flexural strength of 450 ± 53 MPa, elastic modulus of 67.2 ± 1.3 GPa and good bonding ability by hydrofluoric acid etching and silanisation in combination with a required minimum thickness of 1 mm [12–14]. However, in vitro testing is limited in its ability to predict clinical survival [15, 16].

Some available clinical studies have examined the longevity of different types of monolithic lithium disilicate restorations. Nevertheless, the determined survival rates refer primarily to circumferential anterior and posterior crowns [3, 17–23]. Currently, there is little data available on the longevity of lithium disilicate inlays and partial crowns [24]. The purpose of the present study was therefore to retrospectively evaluate the medium- to long-term clinical survival of pressed lithium disilicate (IPS e.max Press, Ivoclar Vivadent, Schaan, Liechtenstein) inlays and partial crowns placed in posterior teeth. In addition, patient satisfaction in terms of aesthetics and function should be determined, and subjective aesthetic outcomes of tooth-coloured restorations should be carried out with the help of intraoral photographs and visual analogue scales (VAS).

## Materials and methods

### Study design and participants

A retrospective clinical study was designed in accordance with the Declaration of Helsinki. The study was conducted in the Department of Conservative Dentistry and Periodontology of the LMU University Hospital in Munich, Germany. The investigation was granted approval by the local ethics committee (Registration number: 20–0919). Patients who received at least one lithium disilicate inlay or partial crown (IPS e.max Press, Ivoclar Vivadent) in the student training program (4th and 5th year of clinical training and boarding exams) in our department between 01.01.2012 and 01.08.2016 were called or contacted by postcard to participate in the study. Patients who were subjected to a wardship were not included. No further exclusion criteria were applied. Before inclusion in the study, every patient had to provide written informed consent.

### Assessment of the restorations and calibration

Relevant data about the tooth and the adhesive-bonding process of the indirect restorations were collected from the patient records before the examination appointment. Additionally, the records were evaluated to analyse any previous complications or failures. Two calibrated dentists (SL and IF) performed the clinical examinations to ensure an objective and reproducible assessment of the ceramic restorations. The calibration of the examiners was achieved through several practice lessons with the study coordinator using standardised cases. Calibration was also achieved by the web-based training tool E-calib [25, 26]. Neither examiner was involved in the earlier treatment procedure of the indirect restorations. If in the process of the independent evaluation of the restorations, there was disagreement among the two investigators, an enforced consensus was needed.

The patients received a general dental examination at the beginning. The lithium disilicate restorations were evaluated according to the FDI criteria as described in detail by Hickel et al. [26, 27]. Aesthetic, functional and biological properties were assessed. The examination was based on the following selected criteria: "surface lustre", "staining surface", "staining margin", "color match and translucency", "aesthetic anatomical form", "fracture of material and retention", "marginal adaption", "occlusal contour and wear qualitatively", "approximal anatomical form (contact point)", "recurrence of caries, erosion, abfraction", "tooth integrity" and "periodontal response" compared to a reference tooth.

A dental mirror, special probes (150 µm, SG5, Deppeler SA, Switzerland), dental floss and metal matrix bands (25-µm, 50-µm, 100-µm width, Deppeler SA, Switzerland) were used in the clinical examination. The sensitivity of the vital restored teeth to cold was evaluated with a cold spray (Orbis Dental, Münster, Germany) and foam pellets. Sensitivity to percussion and tooth mobility was also verified. Two intraoral photographs (occlusal and buccal) were taken of each restoration for further aesthetic evaluation. No radiographs were taken in this study for ethical reasons. The gradings made for each of the selected FDI criteria and the resulting clinical interventions are presented in Table 1.

**Table 1:** Grading and clinical implication

Score	Grading	Clinical implication
1	Clinically very good	No treatment required
2	Clinically good	No treatment required
3	Clinically sufficient/satisfactory	No treatment required
4	Clinically unsatisfactory	Repair possible
5	Clinically poor	Replacement necessary

Evaluated pressed lithium disilicate restorations were both fabricated and placed by dental students. For cementation, the intaglio surface of the restoration was etched with hydrofluoric acid for 20 s (Vita Ceramics Etch 5%, Vita, Bad Säckingen, Germany), rinsed with water spray, air dried and silanised (Monobond Plus, Ivoclar Vivadent). After etching the tooth surface (enamel 30 s, dentine 15 s) with phosphoric acid (Total Etch 37%, Ivoclar Vivadent), restorations were adhesively luted in total etch technique with the three-bottle adhesive system Syntac (Ivoclar Vivadent) along with the dual-core resin system Variolink II (Ivoclar Vivadent). The shade of the adhesive cement was selected by students after try-in of the restorations. The polymerisation time was 40 s at 1100 mW/cm^2^ from each direction (Bluephase Style, Ivoclar Vivadent).

### Patient satisfaction and aesthetic grading

A standardised questionnaire was used to determine patient satisfaction regarding ceramic restorations [28, 29]. Both aesthetics and function could be rated by patients with a score from 1 to 5 (1 = very good, 2 = good, 3 = satisfactory, 4 = sufficient, 5 = deficient).

The intraoral photographs taken in the clinical examination were used for the aesthetic classification of the restorations. The aesthetics of each inlay and partial crown were subjectively evaluated by both clinical investigators and two independent dental technicians using visual analogue scales (VAS). The dental technicians were not involved in the earlier manufacturing process of the restorations. All the images were photographed with a professional single-reflex lens camera (Nikon D7200 with a Nikon Micro 105-mm lens) and Macro Flash MF18 (Nissin Digital) after tooth cleaning and drying. Posterior teeth were photographed indirectly using intraoral mirrors heated before positioning in the oral cavity to prevent condensation on the mirror surface.

### Statistical analysis

Statistical analysis was performed using SPSS software (Version 28.0, SPSS, Chicago, IL, USA). Descriptive statistics were used to illustrate the frequency distributions of the evaluated criteria and to analyse patient satisfaction and aesthetic classification. Kaplan–Meier analysis was applied to determine the survival (scores 1–4) and success (scores 1–3) rates of the investigated restorations. Five-year mean annual failure rate (mAFR) for survival was calculated by the following formula [30].

$${\left(1-y\right)}^{5}=1-x$$

$$y=1-\sqrt[5]{1-x}$$

*y* = 5-year mean annual failure rate; *x* = failure rate

The two-way random intra-class correlation coefficient (ICC) was used to assess the inter-rater agreement among the two calibrated dentists. The following formula for the two-way random effects model was used [31].

$$\frac{MSR- MSE}{MSR+\left(k-1\right)MSE+\left(\frac{k}{n}\right)(MSC-MSE)}$$

MSR = mean square for rows; MSE = mean square for error; MSC = mean square for columns

## Results

### Study sample

A total of 104 patients (56 females, 48 males) ranging in age from 32 to 83 years (median age being 58 years) participated voluntarily in this retrospective study. This accounts for a response rate of 44.3%. A total of 143 indirect IPS e.max Press restorations were assessed. Details on the kind of indirect restoration, tooth type, localisation, endodontic treatments of the restored teeth, the number of restorations per patient/sex and students` experience are shown in Table 2. The mean service time was 5.9 years (71 months) with a minimum observation period of 5.0 years (60 months) and a maximum observation period of 8.3 years (100 months).

**Table 2:** Details on the indirect restorations in the study population

	No. of restorations	Percentage (143 restorations)
Inlay	29	20.3
Partial crown (all cusp coverage with preservation of buccal and lingual/palatal tooth structure)	114	79.7
Premolars	59	41.3
Molars	84	58.7
Premolars maxilla	42	29.4
Premolars mandibula	17	11.9
Molars maxilla	40	27.9
Molars mandibula	44	30.8
Vital restored teeth	103	72.0
Restored teeth with endodontic treatment	38	26.6
Restored teeth with filled access cavities after endodontic treatment (due to irreversible pulpal inflammation)	2	1.4
Restorations in males	61	42.7
Restorations in females	82	57.3
Patients with 1 restoration	68	
Patients with 2 restorations	34	
Patients with 3 restorations	1	
Patients with 4 restorations	1	
Placed by 4th year students	44	30.8
Placed by 5th year students	58	40.6
Placed by students of the final boarding exams	41	28.7

### Clinical performance of the restorations

Restorations evaluated with a score of 4 were designated “repair”, and restorations scored 5 were designated “failure”. The descriptive analysis of the FDI criteria and the distribution of the scores within the included restorations are presented in Table 3. A total of 131 restorations (91.3%) were classified as success (scores 1–3), and 138 restorations (96.5%) were classified as survival (scores 1–4).

**Table 3:** Descriptive analysis of the selected FDI criteria. Not all criteria could always be evaluated for each restoration (missing neighbouring tooth, total loss of restoration)

FDI criteria/score	Total	1	2	3	4	5
A.1. Surface luster	139	66	53	20	0	0
A.2.a Staining surface	139	128	6	5	0	0
A.2.b Staining margin	138	41	75	20	2	0
A.3. Colour match and translucency	139	28	86	25	0	0
A.4. Aesthetic anatomical form	139	68	44	27	0	0
B.5. Fracture of material and retention	139	127	8	4	0	0
B.6. Marginal adaption	138	42	83	12	1	0
B.7.a Occlusal contour and wear (qualitatively)	139	68	50	21	0	0
B.8.a Approximal anatomical form (contact point)	132	102	16	13	0	1
C.12. Recurrence of caries, erosion, abfraction	139	92	31	11	2	3
C.13. Tooth integrity (enamel cracks, tooth fractures)	138	44	81	12	1	0
C.14. Periodontal response	139	45	84	10	0	0

In total, 5 restorations were categorised as failures that exclusively involved partial crowns on molars. Three partial crowns had to be replaced because secondary caries were not accessible for restoration repair. Another reason for failure was a lack of an approximal contact point and resulting damage to the restored tooth due to food impaction. Failure rate due to biological failure was 0.028. One patient participated in the study with a total loss of the restoration, leaving no option for recementation. Failure rate due to material failure was 0.007. More details on the tooth type and time at which the failures occurred are presented in Table 4. The calculated 5-year mAFR for survival was 0.42%.

**Table 4:** Details on the restorations classified as failures

Tooth	Restoration	Root canal filling	Months in situ	Reason for failure
16	Partial crown	No	31	Secondary caries
37	Partial crown	No	47	Secondary caries
26	Partial crown	Yes	64	No approximal contact point
16	Partial crown	No	81	Secondary caries
37	Partial crown	No	unclear	Total loss

**Fig. 1:**
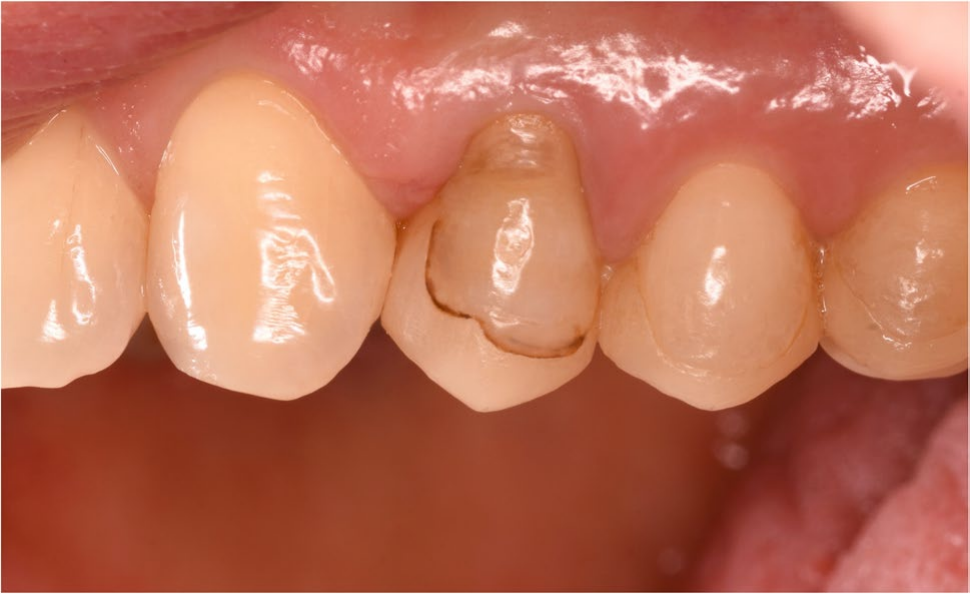
Partial crown 24 with pronounced marginal staining after 65 months

**Fig. 2:**
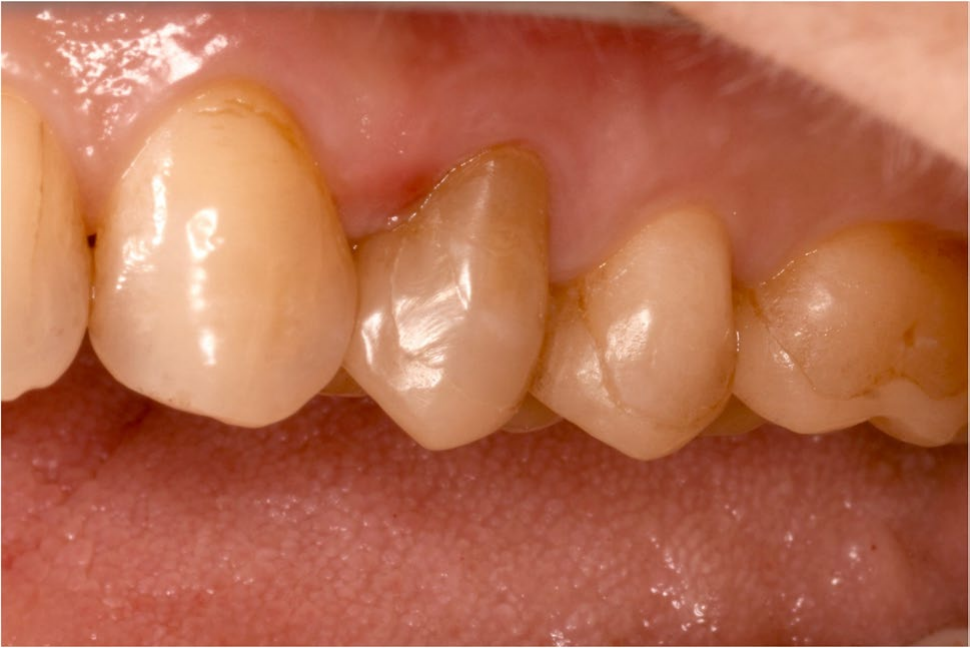
Successful repair of the partial crown 24. Marginal staining was removed by minimally invasive interventions using a very thin bur. The resulting small cavity was filled with flowable composite due to its good rheology

Seven restorations (only partial crowns) were classified as repairs. Reasons for necessary repairs were suspected of undermining caries lesions in 2 cases and debonding in one case. Clinical interventions were necessary to improve the pronounced marginal staining of 2 partial crowns (Figs. 1 and 2). After removing marginal staining and dam application intraoral sandblasting of the ceramic parts and phosphoric etching of the enamel parts (30 s) was performed. Finally, a universal adhesive (Adhese Universal, Ivoclar Vivadent) was applied, polymerised and the cavity was filled with flowable composite (Tetric EvoFlow, Ivoclar Vivadent).

Another 2 restorations were categorised as repairs because of secondary occlusal fillings of access cavities. Both restored teeth required endodontic treatment due to irreversible pulpitis at 21 and 24 months, respectively, after placement of the partial crowns. More details on the tooth type and the service time of the repaired restorations are listed in Table 5.

**Table 5:** Details on the restorations classified as repairs

Tooth	Restoration	Root canal filling	Months in situ	Reason for required repair
15	Partial crown	No	57	Debonding after 36 months
24	Partial crown	Yes	65	Pronounced marginal staining
36	Partial crown	Yes	66	Occlusal access cavity (endodontic treatment)
47	Partial crown	Yes	89	Occlusal access cavity (endodontic treatment)
36	Partial crown	No	90	Pronounced marginal staining
16	Partial crown	No	91	Suspected undermining caries
16	Partial crown	No	98	Suspected undermining caries

**Fig. 3:**
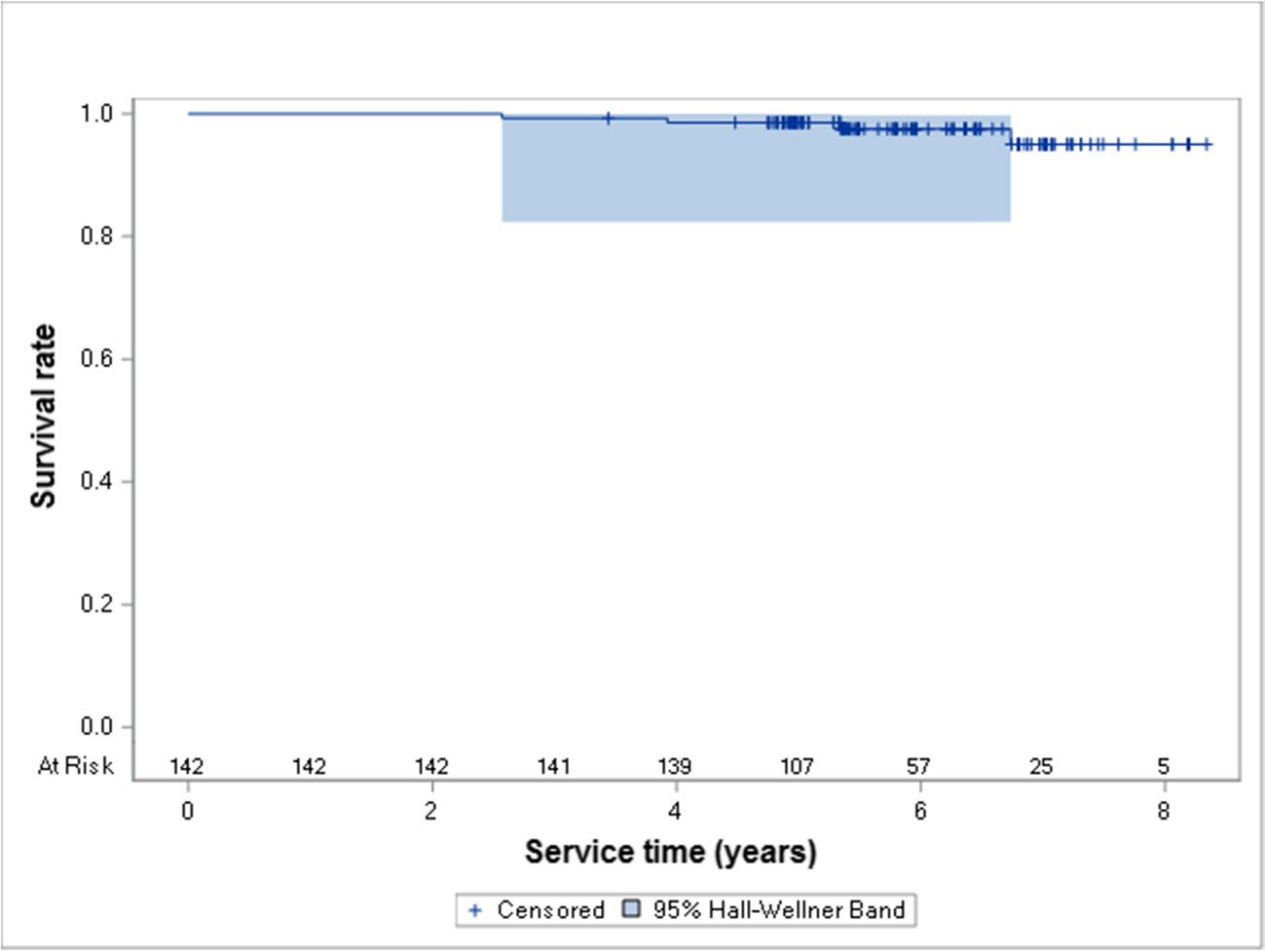
Kaplan–Meier cumulative survival curve for IPS e.max Press restorations in posterior teeth

**Fig. 4:**
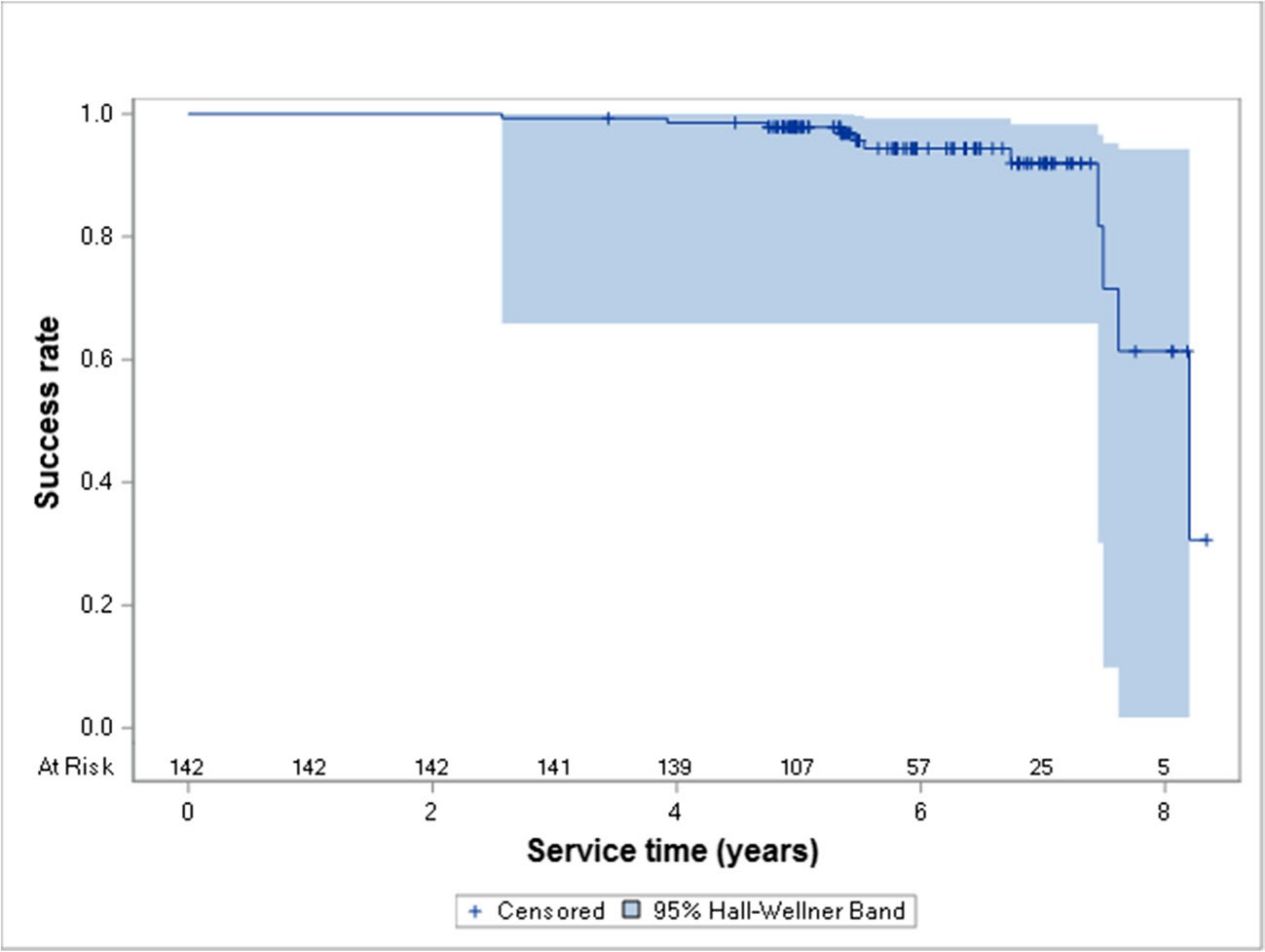
Kaplan–Meier cumulative success curve for IPS e.max Press restorations in posterior teeth

After the mean observation time of 71 months (5.9 years), the cumulative survival and success rates of lithium disilicate restorations in posterior teeth were 97.5% and 94.4%, respectively (Fig. 3 and 4). After 8.3 years, the cumulative survival rate was 95.0%, and the cumulative success rate decreased to 30.7%.

### Inter-rater reliability

ICC between the two independent dentists (inter-rater agreement) was calculated for the overall aesthetic, functional and biological score of the investigated restorations. It was 0.86 for aesthetic properties, 0.93 for functional properties and 0.90 for biological properties.

### Evaluation of the patient questionnaire and VAS

**Fig. 5:**
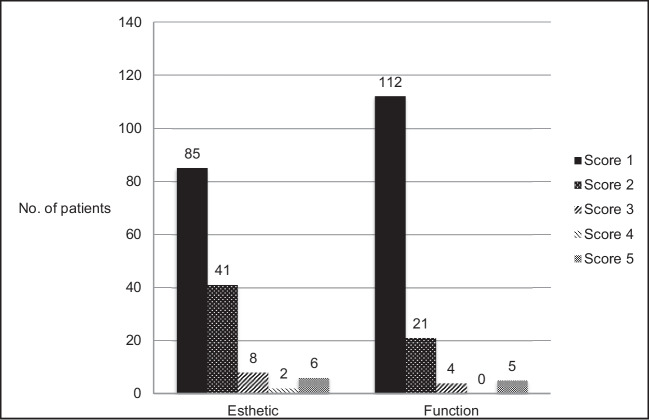
Evaluation of patient satisfaction in terms of aesthetics and function of the indirect restorations. Patient satisfaction of one failure (total loss of the restoration) was not assessed

Assessment of patient satisfaction in terms of aesthetics and function of the restorations is presented in Fig. 5.

**Fig. 6:**
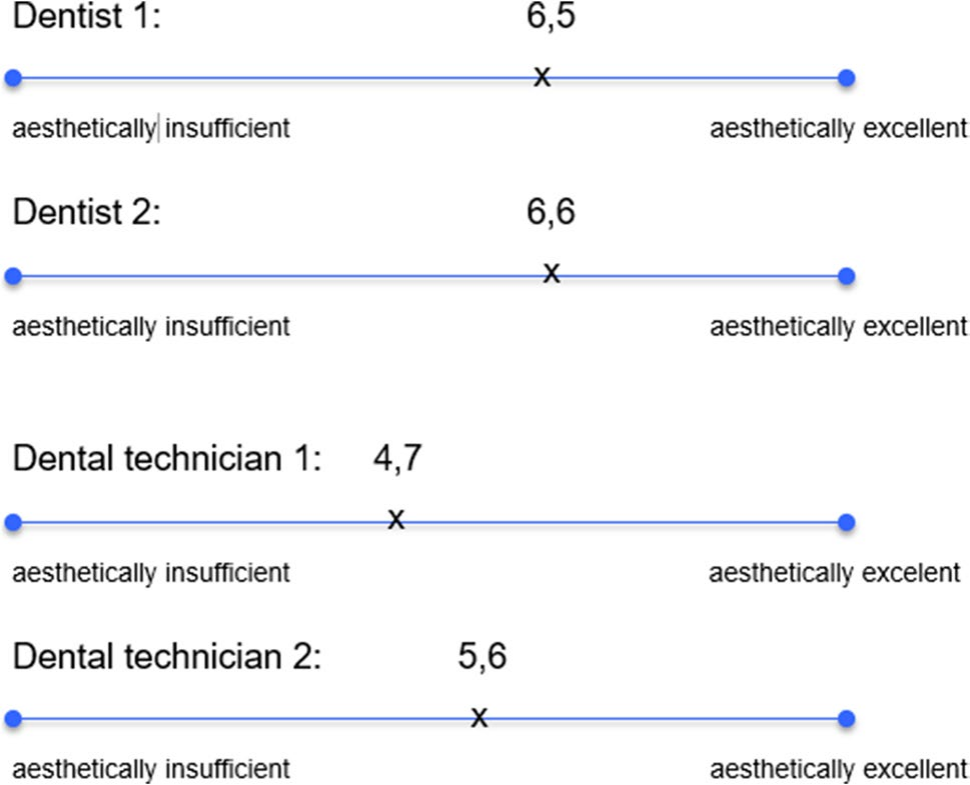
Average values of the subjective evaluation of restorations` aesthetics by two independent dentists and dental technicians

The average subjective rating of the aesthetics of the restorations by the two independent dentists and dental technicians using VAS is illustrated in Fig. 6.

## Discussion

In recent decades, the demand for tooth-coloured restorations in combination with a minimally invasive approach has increased tremendously [2, 32, 33]. For the construction of tooth-coloured inlays and partial crowns in the posterior region, lithium disilicate glass ceramic is currently mainly used. At the time when the indirect restorations of this study were fabricated, IPS e.max was the only glass ceramic recommended, with a reduced minimum thickness of 1 mm [10, 11]. The improved material properties combined with adhesive cementation enable conservative removal of the tooth structure and thus a minimally invasive treatment approach.

Numerous studies have shown high survival rates when restoring single teeth with monolithic lithium disilicate restorations [18, 22–24, 34, 35]. After 10.0 years, survival rates for complete-coverage restorations were calculated to be 83.5%, 85.5% and 96.5%, respectively [18, 22, 23]. In a study by Edelhoff et al. on the clinical performance of minimally invasive occlusal onlays made of lithium disilicate ceramic, the survival rate was 100% after 11.0 years [21]. However, only 7 patients were enrolled in the study. In the present study, a cumulative survival rate of 97.5% and a cumulative success rate of 94.4% after a mean service time of 5.9 years were found for inlays and partial crowns. Comparable results regarding the longevity of lithium disilicate restorations in posterior teeth could be presented in a similar undergraduate student study by Fotiadou et al. where after 6.6 years, the survival rate was calculated to be 96.3% and the success rate to be 93.8% [24]. In line with these results, Archibald et al. evaluated a survival rate of 91.5% for ceramic onlays after 4 years [36].

In total, 5 out of 143 indirect restorations failed in the present study. All failures involved partial crowns on the molars, and the most common reason for failure was secondary caries (*n* = 3). In the study of Archibald et al. 5 restorations were also considered to be failures, and 2 of them needed replacement due to secondary caries [36]. 2 failures caused by secondary caries occurred in the study by Fotiadou et al. [24]. Factors associated with secondary caries risk are patient age, patient socioeconomic status, individual caries susceptibility and localisation of the tooth with a focus on the posterior region [37, 38]. The experience of the operator and skills and care during restoration placement is also important factors impacting restoration longevity and the risk of secondary caries [39–42]. In the current study, the undergraduate students’ low clinical experience level might explain secondary caries as the most common reason for failure.

One of the 5 failures occurred due to the total loss of a partial crown on a molar, leaving no possibility for replacement. The debonding may be attributed to insufficient moisture control during the cementation process or inadequate application of the luting material [43, 44]. In addition, a small amount of enamel available can diminish the bonding strength. Bonding to dentin has not yet reached the ideal characteristics due to the specific properties of dentin, such as its tubular structure and its intrinsic wetness [45]. In the present study, restorations were adhesively luted using a three-bottle etch-and-rinse adhesive system. It is a highly sensitive but particularly effective technique for the cementing of indirect restorations due to its lower risk of hydrolytic degradation at the interface level compared to self-etch strategies [46]. Also in the study of Fotiadou et al. a three-bottle etch-and-rinse system was used and debonding occurred in one case [24]. In contrast, no loss of restoration was reported in a similar clinical investigation in which the restorations were adhesively luted in total etch technique with universal adhesives [36].

In one case in our study, a failure was caused by a missing approximal contact point with accompanying gingivitis of the restored tooth due to food impaction. From the patient record, relevant data about the intraoral fitting of the partial crown and the cementation procedure were analysed, and there was no evidence of an inadequate approximal contact point. However, generalised periodontal disease was noted in the patient´s archive. Tooth movement may be a possible reason for the existing gap between the restored tooth and the neighbouring tooth in this case.

In the comparable clinical study of Fotiadou et al. the most common reason for failure was a bulk fracture in 5 out of 10 failed restorations [24]. Inadequate polishing after occlusal adjustment and the disregard of minimum thickness requirements for lithium disilicate ceramic have been considered explanations for the occurrence of fractures. Archibald et al. reported 3 failed restorations due to fractures caused by insufficient surface polishing after adjustment of the premature occlusal contacts [36]. Fractures and chipping, as known complications in ceramic materials, have also been discussed in other retrospective clinical studies [21, 23, 47–49]. In our investigation, neither chipping nor fractures were detected in total. This result may be attributed to a strict treatment protocol followed in the student training program. Before the full arch impression, the minimum occlusal reduction of the tooth preparation was controlled with a silicon bite to meet minimum thickness requirements for ceramic restorations. Additionally, after cementation, the occlusal contacts were adjusted with diamond finishing rotary instruments under sufficient water cooling and then polished with 3 different porcelain polishing points and a diamond polishing paste to ensure an immaculate surface texture of the restorations. Surface roughness can negatively influence ceramic strength and facilitate crack initiation [50, 51].

7 restorations were classified as repairs. Among these were 2 teeth presenting with suspected undermining caries accessible for repair. Secondary caries is considered a common late complication of restorations and not an early complication, as was the case for both partial crowns in our study (91 and 98 months in situ) [27].

Another 2 restorations on molars were considered repair because of occlusal access cavities prepared for root canal treatment. Both molars required endodontic treatment due to irreversible pulpitis 21 and 24 months after restoration placement, respectively. Access cavities were later filled with composite restorations. These restorations are still in situ and do not affect the marginal adaptation of the lithium disilicate partial crowns and are therefore categorised as repairs. Secondary pulpal complications occurred in a total of 2 among 103 vital restored teeth. In the present investigation, the examiners expected a higher number of pulpal injuries due to the limited clinical experience of the students. Reasons for irreversible pulpal damage might be cavity preparation with large cutting burs, extended contact of the burs with the dentin surface, inadequate cooling and insufficient cleaning of decay due to lack of experience [52]. Comparable frequencies of pulpal complications are found with lithium disilicate restorations when placed by experienced practitioners [5, 18, 22].

In one case in our study, a partial crown on a premolar was considered repair due to debonding of the restoration after 36 months. This partial crown is still in situ 21 months after recementation and is in continuous recall. Several steps must be followed to prepare the tooth and ceramic restoration for successful recementation [53]. Before repeating the adhesive protocol, the residual luting material must be completely removed from the tooth and the internal surface of the restoration. The classic clinical procedure is to remove composite remnants from a lithium disilicate restoration by grinding and sandblasting [54]. The disadvantage of this method is the difficulty of differentiating between luting material and ceramic due to their similar colour. The literature now recommends different methods for removing composite cement, such as laser technology or additional thermal treatment of the restoration [55, 56].

Two partial crowns out of the 7 repairs showed unacceptable marginal staining. Improved aesthetics were achieved through elaborate but minimally invasive clinical interventions using composite (Figs. 1 and 2**)**. Compared to full contour crowns, the position of the preparation margin for partial crowns appears more coronal in the visible area. Marginal staining is, therefore, detectable and impairs the aesthetic appearance of the restoration. However, adhesively luted partial crowns are characterised by a defect-specific, less retentive preparation and allow a reduced loss of hard tooth tissues [57].

The ICCs for inter-rater reliability showed a very good agreement between the two examiners. These findings indicate successful calibration of the two independent dentists using standardised cases and the web-based training tool E-calib. Low inter-rater reliability values of FDI criteria may be obtained if calibration is not performed [58]. However, it is important to note that the authors estimated the inter-rater reliability among only two examiners. The power of any method to detect differences between small groups is very low [59].

In general, the questionnaire results indicate a high level of patient satisfaction. The functionality of the restorations was rated with scores of 1 and 2 more frequently by the participants than the aesthetics. The authors assume this result might be explained by the visible colour difference between the ceramics and tooth structure. The immediate and long term aesthetic result as well as the final colour of lithium disilicate restorations are determined by numerous factors, including the colour of cement and substrate along with the ceramic material itself, being available in various shades and translucency levels [60, 61]. The shade of the tooth stump or a varying layer thickness can influence the aesthetics [62]. The aesthetic outcome of glass ceramic restorations can also be influenced by the shade of the underlying resin composite cement, whereby colour differences are reduced with increasing ceramic layer thickness [61–66]. The low experience level of the students in the colour selection of ceramics and luting material might explain the result of the patient survey.

The subjective aesthetics of the restorations were rated better by the examiners than by the dental technicians. These results confirm the differences in the perception of aesthetics between dentists and dental technicians. The authors assume that dental technicians seem to evaluate the aesthetic outcome of ceramic restorations more critically.

In analysing the results of the present retrospective clinical study, several limitations must be considered. The response rate of patients was only 44.3%. Dropout in clinical studies is common and a potential source of bias in terms of evidence based medicine. It can lead to inaccurate results because it can affect the internal and/or external validity. In addition, evaluated lithium disilicate inlays and partial crowns were placed by undergraduate students under controlled (university) settings. The survival rate of these restorations cannot, therefore, be directly transferred to restorations placed by experienced dentists in private practice settings. In addition, the students had to follow a strict adhesive protocol when bonding glass ceramic restorations, but there were still differences in the process. Relevant data about the adhesive-bonding process were collected from the patient archives before the examination of the restorations, and a rubber dam for moisture control was not placed in every case. Additionally, the patients were treated by different operators (students), resulting in a certain heterogeneity in the treatment procedure. No comparisons to a control group could be made because all patients were treated with the same restorative material. Moreover, further analyses were not performed to show a linkage between the survival or success of the restorations with any covariates due to the low number of statistical events (failures, repairs). Most importantly, the data are based on a retrospective and cross-sectional study, and thus, the clinical performance of indirect restorations up to 8.3 years cannot be compared with the baseline situation. Apart from these limitations, the present study also displays several advantages. All restorations were examined by two independent and calibrated dentists. Therefore, a strong bias in the evaluation of the study restorations can be excluded. Almost no exclusion criteria were applied for the enrolled participants, resulting in a representative study sample. In addition, there was a high number of patients recruited, but with a small number of restorations (mostly 1 or 2 restorations) each. Specific patient characteristics, such as dietary habits, occlusion and functional characteristics might not have influenced the present results. Taken together, based on the analysed data up to 8.3 years, monolithic inlays and partial crowns made of lithium disilicate ceramic exhibited good clinical quality and longevity when placed by supervised undergraduate students.

## Conclusion

Despite the limitations of the present retrospective clinical study, it could be shown that monolithic lithium disilicate (IPS e.max Press) inlays and partial crowns are reliable treatment options in posterior teeth. Pressed lithium disilicate restorations survived successfully up to 8.3 years and were characterised by good clinical quality and aesthetics. In the case of existing partial defects, repairs can avoid complete restoration replacement accompanied by the unnecessary loss of hard tooth tissues.

### Supplementary Information

Below is the link to the electronic supplementary material.

Supplementary file1 (PDF 370 KB)
