# Supplementary material for: Retrospective clinical study on the performance and aesthetic outcome of pressed lithium disilicate restorations in posterior teeth up to 8.3 years
Source: Clin Oral Investig. 2023 Oct 23;27(12):7383–93. doi: 10.1007/s00784-023-05328-0 (PMC10713824; doi:10.1007/s00784-023-05328-0)
Supplement: Supplementary file 1 — Supplementary file1 (PDF 370 KB) [file 784_2023_5328_MOESM1_ESM.pdf]

LMU KLINIKUM
Poliklinik für Zahnerhaltung
und Parodontologie
Prof. Dr. Reinhard Hickel

Befundbogen

Nachuntersuchung von indirekten Restaurationen aus monolithischer Lithiumdisilikatkeramik: Erfolgsrate nach 5 Jahren

Untersuchungsdatum:________________ Eingegliedert am:______________

Zahn:______________________________ Flächen:______________________

Endodontische Therapie: ☐ keine ☐ suffizient ☐ insuffizient

1. Klinische Untersuchung anhand von FDI-Kriterien

A Ästhetische Untersuchungskriterien

**A.1**	**Oberflächenglanz**	
	Score	Beschreibung
	1	Glanz vergleichbar mit Schmelz
	2	1) leicht matt, unauffällig bei Sprechabstand<br>2) einige isolierte Poren
	3	1) matte Oberfläche, aber akzeptabel, wenn mit Speichel benetzt<br>2) viele Poren auf über 1/3 der Füllungsfläche
	4	1) raue Oberfläche, kann nicht durch Speichelfilm maskiert werden, einfache Politur nicht ausreichen, weitere Maßnahmen erforderlich<br>2) Lufteinschlüsse
	5	sehr rau, inakzeptable plaqueretentive Oberfläche

A.2.a	Oberflächenverfärbung	
	Score	Beschreibung
	1	keine Oberflächenverfärbung
	2	geringe Oberflächenverfärbung, leicht durch Politur zu beseitigen
	3	moderate Oberflächenverfärbung, diese ist auch an anderen Zähnen vorhanden, ästhetisch akzeptabel
	4	inakzeptable Oberflächenverfärbung der Restauration, umfangreiche Maßnahmen sind zur Verbesserung nötig
	5	starke Oberflächenverfärbung und/oder tiefere Verfärbung, generalisiert oder lokal, nicht durch Intervention zu verbessern

A.2.b	Randverfärbung	
	Score	Beschreibung
	1	keine Randverfärbung
	2	geringe Randverfärbung, leicht durch Politur zu beseitigen
	3	moderate Randverfärbung, ästhetisch akzeptabel
	4	ausgeprägte Randverfärbung, umfangreiche Maßnahmen sind zur Verbesserung notwendig
	5	tiefe Randverfärbung, nicht durch Intervention zu beheben

A.3	Farbpassung und Transluzenz	
	Score	Beschreibung
	1	gute Farbpassung, keine Abweichung in Schattierung und/oder Transluzenz
	2	geringe Abweichung in Schattierung und/oder Transluzenz
	3	deutliche, allerdings akzeptable Abweichung, welche die Ästhetik nicht beeinträchtigt<br>1) opaker<br>2) transluzenter<br>3) dunkler<br>4) heller
	4	lokalisierte klinische Abweichung, welche korrigiert werden kann<br>1) zu opak<br>2) zu transluzent<br>3) zu dunkel<br>4) zu hell
	5	nicht akzeptabel, Austausch erforderlich

A.4	Ästhetisch anatomische Form	
	Score	Beschreibung
	1	ideale Form
	2	geringe Abweichung von der Norm
	3	Abweichung von der Norm, aber ästhetisch akzeptabel
	4	die Form ist beeinträchtigt und die Ästhetik inakzeptabel, Reparatur erforderlich
	5	die Form ist unbefriedigend und/oder verloren, Reparatur ist nicht möglich, Füllungserneuerung ist notwendig

Gesamtscore ästhetische Eigenschaften:____________________

B Funktionelle Untersuchungskriterien

B.5	**Materialfraktur und Retention**	
	Score	Beschreibung
	1	keine Frakturen oder Risse
	2	kleine Haarrisse
	3	mehrere oder große Haarrisse und/oder Materialabplatzungen, welche die marginale Integrität oder den Approximalkontakt nicht beeinträchtigen
	4	1) Materialabplatzungen, welche die Randqualität/ oder den Approximalkontakt beeinträchtigen<br>2) Massenfraktur mit teilweisem Füllungsverlust (weniger als die Hälfte der Füllung
	5	Füllungsverlust (partiell oder vollständig) oder multiple Frakturen

B.6	**Randadaptation**	
	Score	Beschreibung
	1	harmonisches Bild, keine Unebenheiten tastbar, keine weißen oder verfärbten Linien
	2	1) minimaler Spalt (< 150 µm) tastbar, weiße Linien<br>2) kleine marginale Fraktur, durch Politur zu beseitigen<br>3) geringe Furchen, Absätze/Grate, minimale Unregelmäßigkeiten
	3	1) Spalt (< 250 µm) sondierbar, nicht zu beseitigen<br>2) mehrere kleine marginale Frakturen<br>3) größere Unregelmäßigkeiten, Absätze, Furchen
	4	1) Spalt (> 250 µm) sondierbar (Sonde durchdringt den Spalt) oder Dentin liegt frei<br>2) starke Furchen oder marginale Frakturen<br>3) größere Unregelmäßigkeiten oder Absätze (Reparatur erforderlich)
	5	1) Restauration ist locker, aber in situ<br>2) generalisierte große Spalten oder generalisierte Unregelmäßigkeiten

B.7	**Okklusale Kontur und Abnutzung**<br>**a. qualitativ**<br>**b. quantitativ**	
	Score	Beschreibung
	1	a. physiologische Abrasion, Zahnschmelz entsprechend<br>b. Abrasion entsprechend 80-120% derjenigen des Zahnschmelzes
	2	a. normale Abrasion, nur leicht vom Zahnschmelz abweichend<br>b. 50-80% oder 120-150% Abrasion im Vergleich zur Abrasion des Zahnschmelzes
	3	a. abweichendes Abrasionsausmaß als Schmelz, aber innerhalb der biologischen Grenzen<br>b. < 50% oder 150-300% Schmelz entsprechend
	4	a. Abrasion überschreitet deutlich die normale Schmelzabrasion; oder okklusale Kontaktpunkte sind verloren<br>b. Restauration > 300% Schmelzabrasion oder Antagonist > 300%
	5	a. Abrasion zu stark<br>b. Restauration oder Antagonist > 500% Zahnschmelz entsprechend

**B.8**	**Approximal anatomische Form<br>a. Kontaktpunkt<br>b. Kontur**	
	Score	Beschreibung
	1	a. normaler Kontaktpunkt (Zahnseide oder 25 µm Metallstreifen durchführbar)<br>b. normale Kontur
	2	a. Kontakt leicht zu stark, jedoch keine Nachteile (Zahnseide oder 25 µm Metallstreifen nur mit Druck durchzuführen)<br>b. leicht unzureichende Kontur
	3	a. etwas wenig Kontakt, keine Hinweise auf Schädigung des Zahnes, Gingiva oder parodontaler Strukturen; 50 µm Metallstreifen ist durchführbar)<br>b. sichtbar unzureichende Kontur
	4	a. zu schwach und mögliche Schädigung durch Food Impaction; 100 µm Metallstreifen durchführbar<br>b. mangelhafte Kontur, Reparatur möglich
	5	a. zu schwach und/oder Schädigung durch Food Impaction und/oder Schmerzen/Gingivitis<br>b. mangelhafte Kontur, Austausch nötig

Gesamtscore funktionelle Eigenschaften:___________________

C Biologische Untersuchungskriterien

**C.12**	**Sekundärkaries, Erosion, Abfraktion**	
	Score	Beschreibung
	1	keine Karies oder Sekundärkaries
	2	kleine und lokalisierte<br>1) Demineralisation<br>2) Erosion oder<br>3) Abfraktion
	3	größere Bereiche mit<br>1) Demineralisation<br>2) Erosion oder<br>3) Abrasion/Abfraktion, Dentin liegt nicht frei, nur präventive Maßnahmen erforderlich
	4	1) kavitierte Karies und erwartete unterminierende Karies<br>2) Erosion im Dentin<br>3) Abrasion/Abfraktion im Dentin, lokalisiert und zugänglich, Reparatur möglich
	5	tiefe Karies oder freiliegendes Dentin, nicht zugänglich für Reparatur der Restauration

C.13	**Zahnintegrität (Schmelzrisse, Zahnfrakturen)**	
	Score	Beschreibung
	1	komplette Unversehrtheit
	2	1) kleine marginale Schmelzfrakturen (< 150 µm)<br>2) Haarrisse im Schmelz (< 150 µm)
	3	1) marginaler Schmelzdefekt < 250 µm<br>2) Riss < 250 µm<br>3) Chipping am Schmelz<br>4) multiple Risse
	4	1) große marginale Schmelzdefekte, Spalt > 250 µm oder Dentin oder Boden liegt frei<br>2) große Sprünge > 250 µm, Sonde dringt ein<br>3) großes Chipping am Schmelz oder Fraktur einer Wand
	5	Höcker- oder Zahnfraktur

C.14	**Parodontale Antwort (im Vergleich zu einem Referenzzahn)**	
	Score	Beschreibung
	1	keine Plaque, keine Entzündung, keine parodontalen Taschen
	2	wenig Plaque, keine Entzündung (Gingivitis), keine Bildung parodontaler Taschen<br>1) ohne<br>2) mit Überhängen, Spalt oder unzureichende anatomische Form
	3	PBI weicht 1 Grad im Vergleich zum Referenzzahn ab<br>1) ohne<br>2) mit Überhängen, Spalt oder unzureichende anatomische Form
	4	PBI weicht mehr als 1 Grad im Vergleich zum Referenzzahn ab oder Zunahme der Taschentiefe > 1mm mit Notwendigkeit der Intervention<br>1) ohne<br>2) mit Überhängen, Spalt oder unzureichende anatomische Form
	5	schwere/akute Gingivitis oder Parodontitis<br>1) ohne<br>2) mit Überhängen, Spalt oder unzureichende anatomische Form

Referenzzahn:________

Gesamtscore biologische Eigenschaften:____________________

2. Beurteilung der Patientenzufriedenheit

1. Ästhetik:

☐	☐	☐	☐	☐
sehr gut	gut	befriedigend	ausreichend	ungenügend

2. Funktion:

☐	☐	☐	☐	☐
sehr gut	gut	befriedigend	ausreichend	ungenügend

3. Ästhetisches Grading mittels VAS

(Beurteilung anhand von Fotos)

1. Untersuchender Zahnarzt Nr.1:

mangelhafte Ästhetik/
Restauration im Ganzen
auffällig und unnatürlich

herausragende Ästhetik/
Restauration natürlicher
Zahnhartsubstanz entsprechend

2. Untersuchender Zahnarzt Nr.2:

mangelhafte Ästhetik/<br>Restauration im Ganzen<br>auffällig und unnatürlich	herausragende Ästhetik/<br>Restauration natürlicher<br>Zahnhartsubstanz entsprechend

3. Unabhängiger Zahntechniker Nr.1:

mangelhafte Ästhetik/<br>Restauration im Ganzen<br>auffällig und unnatürlich	herausragende Ästhetik/<br>Restauration natürlicher<br>Zahnhartsubstanz entsprechend

4. Unabhängiger Zahntechniker Nr.2:

mangelhafte Ästhetik/ Restauration im Ganzen auffällig und unnatürlich	herausragende Ästhetik/ Restauration natürlicher Zahnhartsubstanz entsprechend

Name Untersucher 1:________________________________

Name Untersucher 2:________________________________
